# Rapid Analysis of Diagnostic and Antimicrobial Patterns in R (RadaR): Interactive Open-Source Software App for Infection Management and Antimicrobial Stewardship

**DOI:** 10.2196/12843

**Published:** 2019-05-24

**Authors:** Christian Friedemann Luz, Matthijs S Berends, Jan-Willem H Dik, Mariëtte Lokate, Céline Pulcini, Corinna Glasner, Bhanu Sinha

**Affiliations:** 1 Department of Medical Microbiology and Infection Prevention University Medical Center Groningen University of Groningen Groningen Netherlands; 2 Certe Medical Diagnostics and Advice Groningen Netherlands; 3 APEMAC Université de Lorraine Nancy France; 4 Infectious Diseases Department CHRU-Nancy Université de Lorraine Nancy France

**Keywords:** antimicrobial stewardship, software, hospital records, data visualization, infection, medical informatics applications

## Abstract

**Background:**

Analyzing process and outcome measures for all patients diagnosed with an infection in a hospital, including those suspected of having an infection, requires not only processing of large datasets but also accounting for numerous patient parameters and guidelines. Substantial technical expertise is required to conduct such rapid, reproducible, and adaptable analyses; however, such analyses can yield valuable insights for infection management and antimicrobial stewardship (AMS) teams.

**Objective:**

The aim of this study was to present the design, development, and testing of RadaR (Rapid analysis of diagnostic and antimicrobial patterns in R), a software app for infection management, and to ascertain whether RadaR can facilitate user-friendly, intuitive, and interactive analyses of large datasets in the absence of prior in-depth software or programming knowledge.

**Methods:**

RadaR was built in the open-source programming language R, using Shiny, an additional package to implement Web-app frameworks in R. It was developed in the context of a 1339-bed academic tertiary referral hospital to handle data of more than 180,000 admissions.

**Results:**

RadaR enabled visualization of analytical graphs and statistical summaries in a rapid and interactive manner. It allowed users to filter patient groups by 17 different criteria and investigate antimicrobial use, microbiological diagnostic use and results including antimicrobial resistance, and outcome in length of stay. Furthermore, with RadaR, results can be stratified and grouped to compare defined patient groups on the basis of individual patient features.

**Conclusions:**

AMS teams can use RadaR to identify areas within their institutions that might benefit from increased support and targeted interventions. It can be used for the assessment of diagnostic and therapeutic procedures and for visualizing and communicating analyses. RadaR demonstrated the feasibility of developing software tools for use in infection management and for AMS teams in an open-source approach, thus making it free to use and adaptable to different settings.

## Introduction

### Background

With antimicrobial resistance (AMR) on the rise, efforts are being made worldwide to focus on the preservation of antimicrobials as a precious nonrenewable resource. Infection management in the form of antimicrobial stewardship (AMS) programs has emerged as an effective solution to address this global health problem in hospitals. AMS programs are defined as “a coherent set of actions which promote using antimicrobials responsibly” [1]. Stewardship interventions and activities focus on individual patients (personalized medicine and consulting) as well as patient groups or clinical syndromes (guidelines, protocols, information technology infrastructure, and clinical decision support systems) while prioritizing improvement in quality of care and patient safety for any intervention. The appropriate use of antimicrobials based on accurate and timely diagnostics is integral for the successful management of infections. In doing so, the diagnostics contribute to efforts in minimizing AMR by optimizing the use of antimicrobials.

AMS setups in hospitals are often heterogeneous, but audit and feedback to assess the goals are essential parts of most programs, and they are included in international guidelines and reviews [2-7]. Important data for AMS programs include, for example, days of therapy (DOT), daily defined doses (DDD), admission dates, length of stay (LOS), and adherence to local or national diagnostic, therapeutic, or infection management guidelines [1]. Clinical outcomes, quality of care, or consumption of hospital resources can be measured, for example, using mortality data or surrogate parameters such as LOS. The collection of these data is facilitated by electronic health records (EHRs) and administrative local databases. Notably, administrative data have also been shown to be a reliable source for assessing clinical outcomes [8].

EHRs usually offer quick insights into useful infection management data on the individual patient level. However, easy access to analyze patient groups (eg, stratified by departments or wards, specific antimicrobials, or diagnostic procedures used) is difficult to implement in daily practice. It is even more challenging to rapidly analyze larger patient populations (eg, spread over multiple specialties) even though this information might be available. Nevertheless, this is vital for meaningful analysis, including possible confounders and pattern recognition across different populations. Moreover, when aggregated data are available, it is often not possible to trace individual patients, and analyses lack the ability to be further adjusted or stratified.

AMS teams are multidisciplinary, and they act beyond the borders of single specialties [9]. They are usually understaffed, with limited data analysis support [10,11]. Therefore, they need user-friendly and time-saving data analysis resources, without the need for profound technical expertise once the system is set up. Aggregating and linking data of antimicrobial use, guideline adherence, and clinical outcomes at the institutional level can build the basis for important insights for these teams. These could be used to identify areas within hospitals that might benefit most from supportive AMS interventions (eg, subspecialties with lower guideline adherence or unusual patterns of antimicrobial use). Moreover, feedback from these data could help physicians better understand their patient population as a whole; in addition, hospital administration could allocate resources in a more targeted fashion.

Furthermore, aggregated data and simultaneous analysis of multiple areas (eg, use of diagnostics and antimicrobials) present an extensive insight into large patient populations. This also enables the development of comprehensive and multidisciplinary approaches of infection management, combining diagnostic and therapeutic perspectives [1,9,12]. Unfortunately, these kinds of analyses still require substantial statistical knowledge and software skills, and it is time consuming when performed.

Technology, data science, and software app development can bring solutions to complex data handling problems such as those described above. Software app development for medical and epidemiological (research) questions has found many important answers during recent years. For example, software apps at hospital emergency departments (EDs) in the form of a dashboard have been shown to improve efficiency and quality of care for patients requiring emergency admission to hospital [13]. These software apps are used to communicate clearly defined clinical problems, such as mortality ratio, number of cardiac arrests, or readmission rate to the EDs. This has led to a decreased LOS and mortality at the EDs. Others used similar approaches to rapidly and interactively display geographical locations of tuberculosis cases without the need of technical expertise improving the understanding of transmission and detection [14]. Furthermore, data-driven fields such as genomics are front runners in developing new, innovative software apps to handle large datasets, in close collaboration with bioinformatics [15]

It is important to note that all of these abovementioned software apps have been created in an open-source approach. This means that the underlying source code can be easily shared, easily modified, and freely distributed through open repositories, such as GitHub [16], taking open-source software license obligations into account. This facilitates collaboration, quality control through code review, and easy adaptation to many different settings and information technology systems, and this supports the use of advanced data visualization for users with minimal experience in programming and little or no budget for professional database engineers [15].

In the field of medical microbiology, different approaches have been described to interactively work with microbiological diagnostics data and EHRs: electronic antibiograms, centralized resistance analysis, EHR data mining, and clinical decision support systems for AMS are great examples for innovation in the field [17-19]. However, a full open-source approach for software apps working with combined antimicrobials use and diagnostic data of individual patients on the hospital level in the field of infection management is still lacking.

### Objectives

We followed principles of open knowledge [20] to address the need for an interactive, easy-to-use software app that allows users to investigate antimicrobial use, microbiological diagnostic use, and patient outcomes at an institutional (hospital) level. We developed an open-source, Web-based software app—Rapid analysis of diagnostic and antimicrobial patterns in R (RadaR) that can be used for AMS and infection management. This free software app can be run on regular computers or implemented on local or Web-based servers to be accessed through standard Web browsers. The focus user group of this software app is health care professionals involved in AMS (eg, infectious disease specialists, clinical microbiologists, and pharmacists). Although some technical expertise (basic R knowledge) is needed for installation and implementation, the use of RadaR follows usual Web browser user experiences. RadaR enables rapid and reproducible data analysis without extensive previous analysis expertise in a graphically appealing way while being adaptable to different settings. RadaR’s analyses are based on datasets of individual patients. Therefore, aggregated results can also be stripped down, and additional patient features can be investigated. With this software app, we aim at supporting data-driven hospital insights and decision making for actors in the field of AMS in a free, transparent, and reproducible way.

## Methods

For the development of software in an open-source environment, we used the open-source programming language R in conjunction with RStudio version 1.1.463 (RStudio, Inc) [21], an open-source integrated desktop environment for R [22]. Both R and RStudio are free of charge, and they need to be installed for the development and implementation of RadaR. To build RadaR as a Web-based software app, we used the Shiny package for R [23]. Shiny allows R users to build interactive Web apps without extensive knowledge in Web design and its programming languages. The Web apps can be run and hosted on the Web for free [24], as well as on local or cloud-based servers or on personal computers.

The functionality of R can be easily extended by installing additional packages. All packages used for the development of RadaR are listed in Table 1. RadaR is developed in an open-source environment and licensed under GNU General Public License v2.0 [25], giving options to change, modify, and adapt RadaR to both personal and commercial users’ needs while requiring the need to document code changes [25].

RadaR’s calculations and data aggregation are done reactively on the basis of the selection of the user. Single observations on the patient level build the basis for any calculation. RadaR uses common CSV files as input. A total of 3 different data sources are read in RadaR for admission, antimicrobial, and microbiological data, which are merged and transformed upon start. A patient number or study number is used as a unique identifier. All antimicrobial and microbiological data are checked to ascertain whether they fall in the interval of admission dates.

**Table 1:** Required R packages for RadaR.

R package	Minimal version
AMR	0.5.0
data.table	1.11.6
DT	0.4.0
ggridges	0.5.0
lubridate	1.7.4
plotly	4.8.0
qicharts2	0.5.1
rintrojs	0.2.0
shiny	1.1.0
shinyBS	0.61
shinycssloaders	0.2.0
shinydashboard	0.7.0
shinyjs	1.0.0
shinyWidgets	0.4.3
survival	2.42-6
survminer	0.4.3
tidyverse	1.2.1
viridis	0.5.1
zoo	1.8-3

**Table 2:** Input variables for RadaR.

Variable		Detail
**Admission data**		
	adm_end_date	Discharge date^a^
	adm_id	Admission ID
	adm_route	Origin
	adm_start_date	Admission date^a^
	birth_date	Birth date^a^
	death_during_adm	In-hospital death (TRUE/FALSE)
	gender	Gender
	id	Patient ID or study ID
	specialty	General specialty (internal medicine, surgery, and other)
	sub_specialty	Subspecialty
**Antimicrobial data**		
	ab_route	Administration route
	ab_start_date	Start of antimicrobial^a^
	ab_stop_date	Stop of antimicrobial^a^
	atc_code	Fifth level of the World Health Organization Anatomical Therapeutic Chemical (WHO ATC) classification system^b^
	ddd_per_day	Defined daily dose of antimicrobial according to WHO ATC classification system per day^b^
	id	Patient ID or study ID
**Microbiological data**		
	antimicrobial susceptibility testing	Several columns of tested antimicrobial agents (eg, amoxicillin, ciprofloxacin) with resistance results (R/I/S)
	id	Patient ID or study ID
	material	Test material
	mo	Microbial ID (if test=positive)^c^
	specialty	Ordering specialty
	test_date	Test date^a^

^a^YYYY-MM-DD.

^b^As available on the website [31].

^c^As defined by the AMR package for R [30].

The input data should be structured in a dataset format, where each variable is 1 column and each observation is 1 row. This follows the concept of “tidy data,” as defined by Hadley Wickham [26]. Table 2 displays the set of variables underlying RadaR’s functionality. In our setting for the development of RadaR, these variables originated from 3 different data sources: administrative data from the hospital data warehouse, microbiological data from the laboratory information system, and antimicrobial prescription data from the computerized prescriber order entry system. The data preparation and cleaning process are very specific for each data source, dependent on local data standards, and difficult to generalize. Therefore, Table 2 represents the final variables and formats for the analysis and use with RadaR, referring to the “tidy data” concept above and to the tidyverse R package collection for the preparation process [26,27]. Additional variables are calculated and transformed using the packages lubridate and zoo for time points and intervals, and AMR for antimicrobial (group) names, microbial isolate names, first isolate identification, and resistance analysis [28-30]. Microbiological resistance is calculated per antimicrobial substance or as coresistance if more than 1 substance is selected.

RadaR can be used for graphical exploratory data analysis. Differences in LOS are displayed by a Kaplan-Meier curve in conjunction with a log-rank test, using the survminer package [32]. Time trends for number of admissions, antimicrobial consumption, and resistance counts per year, quarter, or month, are visualized in run charts using the qicharts2 package [33]. Nonrandom variation in these run charts is tested using Anhøj’s rules [34].

RadaR has been developed in macOS High Sierra (1.4 GHz, 4 GB RAM), and it was successfully tested in Windows 7 (3.2 GHz, 8 GB RAM) and Linux (Ubuntu 16.04.4 LTS, 3.4 GHz, 12 GB RAM). A running example version has been deployed to shinyapps.io, a publicly available Web hosting service for R Shiny apps [35]. The entire source code of RadaR is freely accessible on GitHub [36]. We intend to integrate suggestions and feedback coming from its users and the R community. RadaR was developed using data of patients admitted to the University Medical Center Groningen, Groningen, the Netherlands. Data were collected retrospectively, and permission was granted by the ethical committee (METc 2014/530). RadaR can be used locally in protected environments or hosted on the Web, provided appropriate measures have been taken to guarantee data protection, depending on national regulations.

## Results

### Overview

We have developed RadaR, a Web-based software app providing an intuitive platform for rapid analysis of large datasets containing information about patients’ admission, antimicrobial use, and results of microbiological diagnostic tests. This software app can help users (ie, AMS team members) find answers to questions, such as “What are the most commonly used antimicrobials at an institution/specialty/department and have they changed over time?,” “Were adequate microbiological diagnostics performed at the start of antimicrobial treatments?,” “What are the most frequent microorganisms found and their resistance patterns in different departments?,” and “Can we identify priority areas within a hospital where antimicrobial or microbiological diagnostic use has the largest room for improvement?”

### Application Design

**Figure 1:**
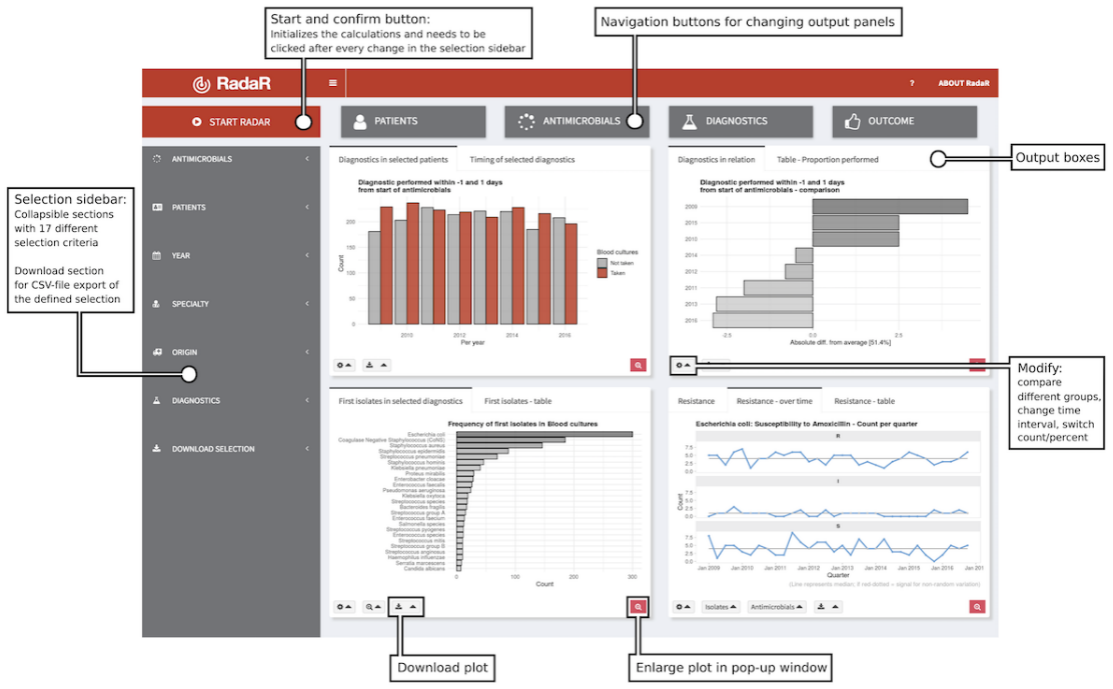
Application design.

RadaR is designed in the form of a Web browser–based dashboard that most users are familiar with from typical websites and Web-based tools (see Figure 1). The basis of RadaR’s functionality is filtering datasets and producing analytical graphs according to selection criteria defined by the user. Any calculations and data aggregation are based on single observations of individual patients. To identify and analyze groups of patients, 17 different selection criteria can be found in the sidebar (Table 3). The output of RadaR is grouped into 4 panels (patient, antimicrobials, diagnostics, and outcome) that each comprise 3 to 4 output boxes displaying the results (see Multimedia Appendix 1).

All output is based on the selection criteria defined by the user in the sidebar. Each new selection and any change need to be confirmed by clicking the confirm selection button (see Figure 1). Users can navigate among the different analysis panels by clicking the respective button.

**Table 3:** Selection criteria in sidebar.

Tab name and criteria		Functionality
**Antimicrobials**		
	Start of antimicrobials (in relation to start of admission)	Select patients starting treatment in a defined time period
	Minimum duration of treatment (days)—all antimicrobials	Select patients with a minimum treatment duration
	Minimum duration of prescription (days)—single antimicrobial	Define the minimum duration of a prescription for any selected antimicrobial
	Administration route	Intravenous or oral
	First antimicrobial only	Filter patients for first prescribed antimicrobial only or any (on the basis of all other selection criteria)
	Groups of antimicrobials	Fourth level of the World Health Organization Anatomical Therapeutic Chemical (WHO ATC) classification system^a^
	Antimicrobials	Fifth level of the WHO ATC classification system^a^
**Patients**		
	Gender	Female or male
	Age	As available in the data
**Year**		
	Year	Years available in the data
**Specialty**		
	Specialty	Internal medicine, surgery, or other
	Minimum number of patients per subspecialty	0, 10, 100, 1000, or 10,000
	Include only this subspecialty	All other subspecialties will be excluded
	Exclude subspecialty	Define single subspecialties to be excluded
**Origin**		
	Origin at admission	As available in the data
**Diagnostics**		
	Type of diagnostics	Blood culture or urine culture test
	Days to first test (in relation to start of antimicrobials)	Define time period for tests to be performed in

^a^As available on the website [31].

Results are shown in bar charts, density plots, run charts, a bubble plot, and a Kaplan-Meier curve for LOS in hospital. Each panel further displays a table summarizing the respective data analyses. All output boxes and their content are described in Table 4. Most output boxes include modification options that can be identified by small gear icons (see Figure 1). These clickable icons allow for further specification of the generated plots and tables. Users can compare different groups (eg, antimicrobial use by antimicrobial agent, resistance patterns per isolate, or LOS by specialty) or modify the plots (eg, switch from count to proportion, change the chart type, or show or hide the legend). Plots and tables can be downloaded through download buttons as PNG files for plots and CSV, Excel, or PDF files for tables.

Finally, 2 datasets (antimicrobial/admission data and microbiological data) of the user-defined selection can be downloaded from the sidebar menu in a CSV-file format for further analysis (eg, retrieving a list of patient numbers of the selected patient group).

**Table 4:** Output boxes for analysis results.

Output panel and output box		Output type	Content	Modification options
**Patients**				
	Subspecialties in selection	Bubble chart^a^	Patients per subspecialty	Show top 10 by number of patients
	Subspecialties—table	Table	Total number of patients and per subspecialty	—^b^
	Patient age	Density plot (distribution)	Age distribution in selection	Group by gender
	Number of admissions	Run chart	Count of admissions per time period	Per year, per quarter, or per month
**Antimicrobials**				
	Antimicrobials	Bar chart	(Group of) antimicrobials sorted by prescription, DDD^c^, or DOT^d^	Single antimicrobials or groups; select prescription count, DDD, or DOT per 100 bed days
	DDD	Run chart	DDD per 100 bed days per group and per time period	Group by none, specialty, subspecialty, and origin; per year, per quarter, and per month
	DOT	Run chart	DOT per 100 bed days per group and per time period	Group by none, specialty, subspecialty, and origin; per year, per quarter, and per month
	DDD/DOT table	Table	Summary of DDD/DOT per 100 bed days per group	DDD or DOT per 100 bed days; group by antimicrobial (group), year, specialty, subspecialty, and origin
**Diagnostics**				
	Diagnostics in selected patients	Bar chart	Diagnostics taken versus not taken in specified timespan	Count or proportion; per year, quarter, or month
	Timing of selected diagnostics	Bar chart	Time of diagnostics performed in days after start of treatment	—^b^
	Diagnostics in relation	Bar chart	Absolute difference from average proportion of selected diagnostics performed	Group by antimicrobial (group), year, specialty, subspecialty, and origin
	Table—proportion performed	Table	Summary of proportion of diagnostics performed	Group by antimicrobial (group), year, specialty, subspecialty, and origin
	First isolates in selected diagnostics	Bar chart	First isolates of microorganisms sorted by frequency	Group by antimicrobial (group), year, specialty, subspecialty, and origin; zoom to select more or less isolates shown in graph
	First isolates—table	Table	Frequency table of first isolates	Group by year, specialty, subspecialty, and origin
	Resistance analysis	Bar chart	Count or proportion of resistance or coresistance to selected antimicrobials in selected isolates in “R,” “S,” and “I” categories	Select isolates; select antimicrobials; group by year, specialty, subspecialty, and origin; select count or proportion
	Resistance—over time	Run chart	Count of resistance or coresistance to selected antimicrobials in selected isolates in “R,” “S,” and “I” categories over time	Select isolates; select antimicrobials; per year, per quarter, or per month
	Table	Table	Count or proportion of resistance or coresistance to selected antimicrobials in selected isolates in “R,” “S,” and “I” categories	Group by year, month, quarter, specialty, subspecialty, and origin; select isolates; select antimicrobials; select count or proportion
**Outcome**				
	Length of stay	Density plot or histogram	Distribution of length of stay per group	Group by all, gender, year, antimicrobial (group), diagnostics performed, specialty, subspecialty, and origin; show histogram; show legend; spread out to remove overlaps
	Length of stay—Kaplan-Meier	Kaplan-Meier curve	Kaplan-Meier curve per group	Groups shown as selected in the length-of-stay box
	Length of stay—table	Table	Summary of length of stay per group	Group by gender, year, antimicrobial (group), diagnostics performed, specialty, subspecialty, and origin

^a^Interactive plot showing additional information when hovering over plot.

^b^Not applicable.

^c^DDD: defined daily doses.

^d^DOT: days of therapy.

### Development Process

RadaR has been developed in close contact with the AMS team and senior consulting specialists at the University Medical Center Groningen, Groningen, the Netherlands, to meet the needs and requirements of this user group. Subsequently, all members of the European Society of Clinical Microbiology and Infectious Diseases Study Group for Antimicrobial Stewardship (ESGAP) were asked to evaluate and test the software app through a running Web-based example of RadaR and by filling out a Web-based survey. The ESGAP comprises around 200 members from more than 30 countries worldwide. A total of 12 members from 9 different countries took part in the evaluation. This yielded important information on user experiences with the software app, which in turn led to further improvements that are reflected in the version we presented in this report. In a next phase, RadaR will be tested in different settings of ESGAP members and other interested partners using locally available data (eg, an 837-bed tertiary care hospital in the Netherlands and a 750-bed tertiary care hospital in Greece).

### Workflow

RadaR was developed and tested with a dataset of all patients admitted to our institution, a 1339-bed academic tertiary referral hospital, within the years of 2009 to 2016, comprising over 180,000 admissions. For simulation purposes and Web-based user testing, we have created a test dataset of 60,000 simulated patients. This sample dataset allows testing of RadaR’s functionality, but it does not produce meaningful results.

A typical example workflow with RadaR comprises 6 steps (with examples from the test dataset). They are listed below:

1. Define the selection: For example, patients receiving intravenous second- or third-generation cephalosporins as first treatment for at least 2 days, starting within the first 2 days of hospital admission from any specialty in all years in the dataset.
2. Patients’ panel: Identify the total number of patients and the subspecialties with the highest number of included patients (eg, 537 patients selected in total, with 97 patients from internal medicine). Investigate patients’ gender and age distribution.
3. Antimicrobials panel: Identify the total use of the initial cefuroxime treatment in DDD and DOT per 100 bed days (eg, 4.51 and 1.5, respectively). Stratify the results by subspecialty and identify the highest number of DDD and DOT per 100 bed days (eg, highest use by DDD and DOT in internal medicine).
4. Diagnostics panel: Check if the selected microbiological diagnostic test (eg, blood culture test) has been performed on the same day as the start of the treatment (defined in the sidebar). Investigate the proportion of tests performed over the years and investigate which subspecialty performs best compared with others (eg, Pediatrics). Check which microorganisms (as first isolates) were found in the selected diagnostic specimens (the most common isolate: *Escherichia coli*). Investigate the proportion of isolates resistant to cefuroxime (8.9%) and analyze the trend over time.
5. Outcome panel: Check for patterns of differences in LOS in the defined patient group by subspecialties or performed diagnostics (eg, highest mean LOS of 7.8 days in Surgery).
6. Refine the selection: Investigate a subgroup of the original selection. For example, select only the top 3 subspecialties by number of patients and repeat step 2 to 5.

### Customization

For setting up RadaR in a new environment after data preparation, users only need to perform the following 4 steps:

1. Downloading R and RStudio [21,22], which are free to use and open-source software
2. Download or copy and paste RadaR’s source code [36] into 3 files in RStudio—global.R, server.R, and ui.R
3. In global.R, manually edit the paths for the prepared datasets to be imported into RadaR
4. Run the app in RStudio with the calling the function runApp() in the console or by clicking the green run app button. This will download and install the required R packages needed for the app if they have not been installed previously, and this will create the final dataset for analysis. The RadaR interface will open in the RStudio viewer pane or in a new window of the standard browser of the user’s operating system.

RadaR’s appearance has been customized using a cascading style sheets (CSS) script [37] that is loaded into the app upon its start. This script needs to be saved into a subdirectory of the directory of the 3 main files (global.R, server.R, and ui.R) called “www.” We recommend RStudio’s project function to create a single project for RadaR and to store all information in this project directory. Users with experience in using CSS can fully alter RadaR’s design by changing the underlying CSS script.

## Discussion

### Principal Findings

We have developed a Web-based software app for rapid analysis of diagnostic and antimicrobial patterns that can support AMS teams to tailor their interventions. It has been designed to enhance communication of relevant findings while being easy to use. This also applies to users without extensive prior software skills, as it follows usual Web browser user experiences. Moreover, it has been developed using open-source software. It is therefore free to use and accessible for download. In our experience, this system can be adapted to new settings within 1 day, when the required data (Table 2) are available.

Commercial software for infection management is available (eg, Epic Antimicrobial Stewardship Module, TREAT Steward). These offer extensive options for filtering, analyzing, and visualizing EHRs with real-time connections to hospital data infrastructures and have been shown to be useful in clinical practice [38]. However, it is difficult to compare functionalities of these tools because of their non–open-source nature. This fact, along with the required budget to purchase the software, drastically limits their use. We are convinced that transparent software development can support the adoption of data-driven developments while enhancing optimal quality of care and patient safety, which is crucial in the light of new data-driven developments of using EHRs [39,40].

The global nature of infections further calls to develop software tools applicable in resource-limited settings [41]. Open-source approaches for data analysis, such as RadaR, have advantages over traditional methods, such as Excel or SPSS. Hughes et al described those in their report of a software app for RNA-sequencing data analysis [15]. They highlight aspects that were also fundamental for the development of RadaR. First, R allows transparent, reproducible, and sustainable data analysis through scripts that can easily be shared and changed. This can build the basis for collaboration, and this enforces the spirit of open science (also through the strong collaborative R community on the Web). Second, R is open source and free to use; therefore, it also enables use in resource-limited settings. Finally, Shiny empowers users to interact with the data, making even very large datasets quickly interpretable.

Innovative approaches used in supporting infection management by leveraging EHRs are being investigated [17-19]. Reporting on AMR, antimicrobial use, and hospital infections (eg, for quality assurance) is well established, but it is important to integrate these data sources in an approach that allows detailed filtering options on all input. Merely looking at antimicrobial use alone or comparing aggregated results (eg, total amount of a specific antimicrobial substance per hospital correlated with the total count of a resistant isolate) will result in loss of information or even misleading interpretation. Detailed data and calculations on the basis of each individual patient are crucial to draw informed conclusions. Unfortunately, the abovementioned infection management approaches [17-19] either depend on additional commercial software for data visualization or the source code is not openly available. We want to encourage others to turn toward available open-source software solutions, such as R, for an increased potential of collaboration and transparency. However, their strength is the connection to real-time data flows. This enables the prospective use and increases their usability for daily clinical practice. RadaR is currently still limited to retrospective data analysis because of a changing hospital data infrastructure in our setting. Technically, it is feasible to connect R-based software apps such as RadaR to real-time hospital data infrastructures running with clinical data standards [42]. For a start, access to static data extraction is often easier and faster to achieve. RadaR can be used to advocate the use of data visualization tools and improved accessibility of hospital data sources. Until connection to real-time hospital data is established, RadaR can support users as a stand-alone option for retrospective data analysis in infection management. Next steps will involve testing in multiple settings and forming a user and research group to continue and expand the use of open-source technology and open science principles in infection management.

### Conclusions

RadaR demonstrates the feasibility of developing software tools for infection management and AMS teams in an open-source approach, making it free to use, share, or modify according to various needs in different settings. RadaR has the potential to be a highly useful tool for infection management and AMS in daily practice.

## Appendix

Multimedia Appendix 1 Screenshot of RadaR.

## Abbreviations

AMR: antimicrobial resistance
AMS: antimicrobial stewardship
CSS: cascading style sheets
DDD: daily defined doses
DOT: days of therapy
EDs: emergency departments
EHR: electronic health record
ESGAP: European Society of Clinical Microbiology and Infectious Diseases Study Group for Antimicrobial Stewardship
LOS: length of stay
RadaR: Rapid analysis of diagnostic and antimicrobial patterns in R

## References

[1] Dyar OJ, Huttner B, Schouten J, Pulcini C, ESGAP (ESCMID Study Group for Antimicrobial stewardshiP) (2017). What is antimicrobial stewardship?. Clin Microbiol Infect.

[2] Barlam TF, Cosgrove SE, Abbo LM, MacDougall C, Schuetz AN, Septimus EJ, Srinivasan A, Dellit TH, Falck-Ytter YT, Fishman NO, Hamilton CW, Jenkins TC, Lipsett PA, Malani PN, May LS, Moran GJ, Neuhauser MM, Newland JG, Ohl CA, Samore MH, Seo SK, Trivedi KK (2016). Implementing an Antibiotic Stewardship Program: guidelines by the Infectious Diseases Society of America and the Society for Healthcare Epidemiology of America. Clin Infect Dis.

[3] Davey P, Marwick CA, Scott CL, Charani E, McNeil K, Brown E, Gould IM, Ramsay CR, Michie S (2017). Interventions to improve antibiotic prescribing practices for hospital inpatients. Cochrane Database Syst Rev.

[4] National Institute for Health and Care Excellence.

[5] Schuts EC, Hulscher ME, Mouton JW, Verduin CM, Stuart JW, Overdiek HW, van der Linden PD, Natsch S, Hertogh CM, Wolfs TF, Schouten JA, Kullberg BJ, Prins JM (2016). Current evidence on hospital antimicrobial stewardship objectives: a systematic review and meta-analysis. Lancet Infect Dis.

[6] (2016). Stichting Werkgroep Antibiotica Beleid.

[7] Pulcini C, Binda F, Lamkang AS, Trett A, Charani E, Goff DA, Harbarth S, Hinrichsen SL, Levy-Hara G, Mendelson M, Nathwani D, Gunturu R, Singh S, Srinivasan A, Thamlikitkul V, Thursky K, Vlieghe E, Wertheim H, Zeng M, Gandra S, Laxminarayan R (2018). Developing core elements and checklist items for global hospital antimicrobial stewardship programmes: a consensus approach. Clin Microbiol Infect.

[8] Sarkies MN, Bowles K, Skinner EH, Mitchell D, Haas R, Ho M, Salter K, May K, Markham D, O'Brien L, Plumb S, Haines TP (2015). Data collection methods in health services research: hospital length of stay and discharge destination. Appl Clin Inform.

[9] (2018). British Society for Antimicrobial Chemotherapy.

[10] Pulcini C, Morel CM, Tacconelli E, Beovic B, de With K, Goossens H, Harbarth S, Holmes A, Howard P, Morris AM, Nathwani D, Sharland M, Schouten J, Thursky K, Laxminarayan R, Mendelson M (2017). Human resources estimates and funding for antibiotic stewardship teams are urgently needed. Clin Microbiol Infect.

[11] Howard P, Pulcini C, Levy Hara G, West RM, Gould IM, Harbarth S, Nathwani D, ESCMID Study Group for Antimicrobial Policies (ESGAP), ISC Group on Antimicrobial Stewardship (2015). An international cross-sectional survey of antimicrobial stewardship programmes in hospitals. J Antimicrob Chemother.

[12] Dik JH, Poelman R, Friedrich AW, Panday PN, Lo-Ten-Foe JR, van Assen S, van Gemert-Pijnen JE, Niesters HG, Hendrix R, Sinha B (2016). An integrated stewardship model: antimicrobial, infection prevention and diagnostic (AID). Future Microbiol.

[13] Staib A, Sullivan C, Jones M, Griffin B, Bell A, Scott I (2017). The ED-inpatient dashboard: Uniting emergency and inpatient clinicians to improve the efficiency and quality of care for patients requiring emergency admission to hospital. Emerg Med Australas.

[14] Smith CM, Hayward AC (2016). DotMapper: an open source tool for creating interactive disease point maps. BMC Infect Dis.

[15] Hughes LD, Lewis SA, Hughes ME (2017). ExpressionDB: an open source platform for distributing genome-scale datasets. PLoS One.

[16] GitHub.

[17] Simpao AF, Ahumada LM, Larru Martinez B, Cardenas AM, Metjian TA, Sullivan KV, Gálvez JA, Desai BR, Rehman MA, Gerber JS (2018). Design and implementation of a visual analytics electronic antibiogram within an electronic health record system at a tertiary pediatric hospital. Appl Clin Inform.

[18] Lesho EP, Waterman PE, Chukwuma U, McAuliffe K, Neumann C, Julius MD, Crouch H, Chandrasekera R, English JF, Clifford RJ, Kester KE (2014). The antimicrobial resistance monitoring and research (ARMoR) program: the US Department of Defense response to escalating antimicrobial resistance. Clin Infect Dis.

[19] Simões AS, Maia MR, Gregório J, Couto I, Asfeldt AM, Simonsen GS, Póvoa P, Viveiros M, Lapão LV (2018). Participatory implementation of an antibiotic stewardship programme supported by an innovative surveillance and clinical decision-support system. J Hosp Infect.

[20] Molloy JC (2011). The Open Knowledge Foundation: open data means better science. PLoS Biol.

[21] RStudio.

[22] R Core Team R: A Language and Environment for Statistical Computing.

[23] Chang W, Cheng J, Allaire JJ, Xie Y, McPherson J The Comprehensive R Archive Network.

[24] shinyapps.io.

[25] Stallman R (1991). GNU General Public License, version 2.

[26] Wickham H (2014). Tidy data. J Stat Soft.

[27] Wickham H (2017). tidyverse: Easily Install and Load the 'Tidyverse'.

[28] Grolemund G, Wickham H (2011). Dates and times made easy with lubridate. J Stat Soft.

[29] Zeileis A, Grothendieck G (2005). zoo: S3 infrastructure for regular and irregular time series. J Stat Soft.

[30] Berends MS, Luz CF, Glasner C, Friedrich AW, Sinha B GitLab.

[31] (2018). WHO Collaborating Centre for Drug Statistics Methodology.

[32] Kassambara A, Kosinski M (2018). The Comprehensive R Archive Network.

[33] Anhoej J (2018). The Comprehensive R Archive Network.

[34] Anhøj J (2015). Diagnostic value of run chart analysis: using likelihood ratios to compare run chart rules on simulated data series. PLoS One.

[35] RadaR shinyapps.io.

[36] RadaR GitHub.

[37] RadaR CSS GitHub.

[38] Pettit N, Han Z, Choksi A, Bhagat P, Pisano J (2017). Using the Epic® Antimicrobial Stewardship (ASP) module to optimize antimicrobial stewardship interventions. Open Forum Infect Dis.

[39] Xiao C, Choi E, Sun J (2018). Opportunities and challenges in developing deep learning models using electronic health records data: a systematic review. J Am Med Inform Assoc.

[40] Pirracchio R, Cohen MJ, Malenica I, Cohen J, Chambaz A, Cannesson M, Lee C, Resche-Rigon M, Hubbard A, ACTERREA Research Group (2018). Big data and targeted machine learning in action to assist medical decision in the ICU. Anaesth Crit Care Pain Med.

[41] Hahn E, Blazes D, Lewis S (2016). Understanding How the. Health Secur.

[42] Hong N, Prodduturi N, Wang C, Jiang G (2017). Shiny FHIR: an integrated framework leveraging Shiny R and HL7 FHIR to empower standards-based clinical data applications. Stud Health Technol Inform.

